# Xuebijing improves inflammation and pyroptosis of acute lung injury by up-regulating miR-181d-5p-mediated SPP1 inactivation

**DOI:** 10.1016/j.clinsp.2024.100336

**Published:** 2024-02-06

**Authors:** XiaoYong Wu, RuoMei Xin, YanZhong Zhang, ChengRui Yang, FangYuan Sun, YanLiang Wang, FengXian Zheng

**Affiliations:** a Department of General Surgery, Affiliated Danzhou People's Hospital of Hainan Medical University, Danzhou City, Hainan Province, China; b Department of Nursing, Affiliated Danzhou People's Hospital of Hainan Medical University, Danzhou City, Hainan Province, China; c Department of Critical Care Medicine, Affiliated Danzhou People's Hospital of Hainan Medical University, Danzhou City, Hainan Province, China

**Keywords:** Xuebijing, miR-181d-5p, SPP1, Acute lung injury

## Abstract

**Background:**

Xuebijing (XBJ) is widely applied in the treatment of Acute Lung Injury (ALI). This study focused on the potential mechanism of XBJ in Lipopolysaccharide (LPS)-induced ALI.

**Methods:**

The rat ALI model was established by injection of LPS (10 mg/kg) and pretreated with XBJ (4 mL/kg) three days before LPS injection. BEAS-2B cell line was stimulated with LPS (1 μg/mL) and ATP (5 mM) to induce pyroptosis, and XBJ (2 g/L) was pretreated 24h before induction. The improvement effects of XBJ on pulmonary edema, morphological changes, and apoptosis in ALI lung tissue were evaluated by lung wet/dry weight ratio, HE-staining, and TUNEL staining. Inflammatory cytokines in lung tissue and cell supernatant were determined by ELISA. pyroptosis was detected by flow cytometry. Meanwhile, the expressions of miR-181d-5p, SPP1, p-p65, NLRP3, ASC, caspase-1, p20, and GSDMD-N in tissues and cells were assessed by RT-qPCR and immunoblotting. The relationship between miR-181d-5p and SPP1 in experimental inflammation was reported by dual luciferase assay.

**Results:**

XBJ could improve inflammation and pyroptosis of ALI by inhibiting contents of inflammatory cytokines, and levels of inflammation- and pyroptosis-related proteins. Mechanistically, XBJ could up-regulate miR-181d-5p and inhibit SPP1 in ALI. miR-181d-5p can target the regulation of SPP1. Depressing miR-181d-5p compensated for the ameliorative effect of XBJ on ALI, and overexpressing SPP1 suppressed the attenuating effect of XBJ on LPS-induced inflammation and pyroptosis.

**Conclusion:**

XBJ can regulate the miR-181d-5p/SPP1 axis to improve inflammatory response and pyroptosis in ALI.

## Highlights

• XBJ improves LPS-induced lung cell inflammation and pyroptosis.
• miR-181d-5p inhibits LPS-induced inflammatory response and pyroptosis of lung epithelial cells.
• XBJ elevates miR-181d-5p and improves LPS-induced pyroptosis of lung epithelial cells.
• XBJ upregulates miR-181d-5p and inhibits SPP1 to protect lung epithelial cells from LPS-induced injuries.

## Introduction

Acute Lung Injury (ALI) refers to a series of lung lesions caused by multiple lung injuries, which can induce serious lung diseases and lead to serious sequelae and high mortality [1,2]. ALI is characterized by severe acute inflammatory processes, leading to increased alveolar permeability, protein and white blood cell accumulation, and pulmonary edema [3]. There is still a lack of effective drugs to control and treat ALI [4,5]. Therefore, there is an urgent need to find new drugs to relieve ALI.

Xuebijing (XBJ) is a Chinese herbal compound that is mainly composed of Honghua (Carthamus tinctorius), Chi shao (Paeoniae radix), Danshen (Salvia divinorum), It consists of Danggui (Angelica sinensis) and Chuanxiong (Ligusticum wallichii Franchet) [6]. Reports have emphasized the anti-endotoxin and anti-inflammatory effects of XBJ 7, 8, 9. Some studies have confirmed that XBJ can ameliorate lung injury. For example, XBJ can ameliorate inflammation of lung injury [10]. Recently, XBJ has been approved for the treatment of sepsis in China through clinical trials [11]. However, the detailed mechanism of XBJ in ALI is unclear.

NLRP3-mediated pyroptosis of macrophages can aggravate lung inflammation in patients with ALI [12]. NLRP3 inflammasome activation in alveolar macrophages leads to the processing of pro-caspase-1 into two lysed subunits named p10 and p20, which can induce the release of pro-inflammatory cytokines and trigger severe inflammatory responses [2,13]. However, the association between XBJ's protection of lung tissue and NLRP3-mediated pyroptosis remains unclear. miRNAs can negatively regulate gene expression at the post-transcriptional level [14] and are substantially implicated in inflammatory lung diseases, including ALI. For example, miR-181a inhibition protects mice from ALI [15]. Recent studies have confirmed that miR-181d-5p exerts an anti-inflammatory role after renal ischemia-reperfusion injury and can inhibit the expression of inflammatory mediators, thus improving renal function [16].

SPP1, also known as Osteopontin (OPN), is a coding protein located in 4q22.1 and is considered to be a key cytokine involved in immune cell recruitment and expression of type 1 cytokines at inflammatory sites [17,18]. Current studies have confirmed that SPP1 is involved in tumor cell progression 19, 20, 21, 22. However, its underlying mechanism in inflammation-related ALI has not been fully elucidated.

Here, rat and cellular ALI models were constructed using LPS to explore the potential mechanism of XBJ to improve ALI. At the same time, the effects of XBJ on inflammatory response and pyroptosis in ALI were discussed, as well as the mechanism of miR-181d-5p and SPP1.

## Materials and methods

### ALI animal model

This study was approved by the Animal Protection Professional Committee of Affiliated Danzhou People's Hospital of Hainan Medical University. Male SPF-grade SD rats (180‒220g) were purchased from the Animal Experimental Center, Tongji Medical College, Huazhong University of Science and Technology (Wuhan, China). Rats were kept on a day/night 12/12h cycle with 55% humidity, with free access to water and food. After one week of adaptive feeding, the rats were randomly divided into 3 groups (6 rats/group): Control, ALI, and ALI+XBJ. Rats were first anesthetized by intraperitoneal injection of pentobarbital sodium at 50 mg/kg, followed by intraperitoneal injection of 10 mg/kg LPS (Sigma-Aldrich; USA), while rats in the Control group were injected intraperitoneally with 0.9% normal saline [23]. Three days before modeling, rats in the ALI+XBJ group were injected with XBJ (4 mL/kg) twice a day via caudal vein, while rats in the Control and ALI groups were injected with 0.9% normal saline [24]. All rats were euthanized 12h after LPS injection. Subsequently, the right lung of 3 rats in each group was ligated, and the left lung was rapidly frozen at -80°C for subsequent RNA, protein, and flow cytometry tests. The remaining right lung was fixed in 4% paraformaldehyde for histological analysis. From the remaining 3 rats in each group, lung tissues were weighed first and then dried at 65°C for 48h to calculate the Wet/Dry (W/D) ratio to estimate pulmonary edema index.

### ELISA

Cell culture supernatant or rat lung tissues were centrifuged at 500 × g at 4°C for 10-min for ELISA. Inflammatory cytokines were detected using commercial ELISA kits for IL-1β, IL-18, and TNF-α (R&D System, USA).

### Cell culture and treatment

BEAS-2B Cell line (ATCC) was cultured in DMEM supplemented with 10% FBS and 1% penicillin/streptomycin in a 37°C incubator with 5% CO_2_. XBJ group was pretreated with 2 g/L XBJ (Tianjin Datong New Pharmaceutical Co., LTD., China) in BEAS-2B cells for 24h [25] and treated with 1 μg/mL LPS (Sigma-Aldrich) for 4h and then with 5 mM ATP (A6559, Sigma-Aldrich) for 30 min to induce pyroptosis, while Control and LPS groups were added with equal amounts of PBS [26]. The remaining groups were transfected before treatment with XBJ, LPS, and ATP.

### Cell transfection

miR-181d-5p mimic/inhibitor and oe-SPP1, and their corresponding controls (mimic NC, inhibitor NC, and vector) were synthesized in GenePharma (Shanghai, China) and transfected into BEAS-2B cells using Lipofectamine 2000 (Invitrogen). The medium was replaced at 8h, and cells were harvested at 48h to evaluate the transfection efficiency by RT-qPCR or immunoblotting.

### Immunoblotting

Lung tissue and BEAS-2B cells were lysed with RIPA buffers containing PMSF or phosphatase inhibitors. Protein concentration was detected using a BCA test kit. The equal-volume proteins were separated by 12% SDS-PAGE, transferred to PVDF membrane, blocked with 5% skim milk for 2h, and rinsed with TBST 3 times (10 min/time). Then, NLRP3 (19771-1-AP, Proteintech, USA), Caspase-1 p20 (22915-1-AP, Proteintech), ASC (10500-1-AP, Proteintech), GSDMD-N (ab219800, Abcam, USA), p-p65 (3033, CST), GAPDH (2118, CST), and SPP1 (sc-21742, Santa Cruz Biotechnology) were separately added to incubate overnight at 4°C. Then, the HRP conjugate secondary antibody was supplemented for 1h before visualization of protein bands based on an enhanced chemiluminescence kit (Vazyme, China).

### RT-qPCR

Total RNA was extracted from rat lung tissue and BEAS-2B cells by Trizol Reagent (Invitrogen), and RNA quality and concentration were determined by Nanodrop 2000. Reverse transcription of miRNA was implemented by Taqman® MicroRNA Reverse Transcription kit (Invitrogen), while that of mRNA was done by cDNA synthesis kit (Thermo Fisher Scientific). RT-qPCR assay was performed using the SYBR Premix Ex Taq^TM^ II kit (RR820A, Takara) and analyzed by Biosystems 7900 thermocycler (Thermo Fisher Scientific). With U6 and GAPDH as the reference genes, respectively, expression was calculated by the 2^−ΔΔCt^ method. The primer sequence is shown in Table 1.

**Table 1:** PCR primer sequences.

Genes	PCR primer sequences (5’–3’)
miR-181d-5p	Forward: GCTGAACATTCATTGTTGTCG
	Reverse: GCAGGGTCCGAGGTATTC
SPP1 (Rat)	Forward: TGGATGAACCAAGCGTGGAA
	Reverse: TTTGGAACTCGCCTGACTGT
SPP1 (Human)	Forward: CACATCCCGAGGAGACACAG
	Reverse: GGGCCCAGCTAAAGGTAATGT
U6	Forward: CTCGCTTCGGCAGCACA
	Reverse: AACGCTTCACGAATTTGCGT
GAPDH	Forward: CACCCACTCCTCCACCTTTG
	Reverse: CCACCACCCTGTTGCTGTAG

Note: miR-181d-5p, microRNA-181d-5p; SPP1, Secreted Phosphoprotein 1; GAPDH, Glyceraldehyde-3-Phosphate Dehydrogenase.

### Flow cytometry

BEAS-2B cells were washed twice with pre-cooled PBS and resuspended with 1 mL of 1 × buffer. Then, cells (1 × 10^6^ cells/mL) were mixed with FLICA® 660 Caspase-1 Assay regent (Immunochemistry Technologies, USA) at 37°C for 30-min, added with PI for 5-min, and analyzed using BD FACSAria flow cytometry (BD Company, USA). Caspase-1^+^/PI^+^ cells were pyroptosis cells [27].

### Luciferase reporter assay

miR-181d-5p and SPP1 binding sites were analyzed in the starbase 3.0 (https://starbase.sysu.edu.cn/). WT-SPP1 and MUT-SPP1 constructs were produced by inserting the 3′UTR sequences of wild and mutant SPP1 into the pmirGLO vector (Promega, USA), which were then transfected with miR-181d-5p mimic or mimic NC into BEAS-2B cells using Lipofectamine 2000 (Invitrogen). Luciferase activity after 48h was then assessed using a dual luciferase reporter assay kit (Promega) and recorded on the Synergy 2 Multidetector Microplate Reader (BioTek Instruments).

### HE-staining

The lung tissues were prepared into slices with 5 μm thickness using a microtome (RM2235, Leica, Germany) after fixation in 4% paraformaldehyde for 24h and embedment in paraffin. Then, the tissues were reacted with hematoxylin (Beyotime, China) for 5‒10 min, rinsed with running water for 3-min, dyed with eosin (Beyotime) for 1‒2 min, and viewed using an optical microscope (Olympus).

### TUNEL staining

To measure apoptosis, a TUNEL assay was carried out using a commercial kit (Beyotime). Paraffin sections (5 μm) were incubated with 45 μL labeled buffer and 5 μL TdT enzyme solution for 60-min, rinsed 3 times with PBS, stained with DAPI for 5-min, and observed under a fluorescence microscope (Olympus). Quantification of images was performed with ImageJ software.

### RIP assay

Magna RIP Kit (Millipore) was purchased to conduct RIP assay. Cell lysates were harvested using RIP lysis buffer and combined with magnetic beads with Ago2 or IgG antibodies at 4°C for 6h. After elution, the immunoprecipitates were collected for RT-qPCR analysis.

### Statistical analysis

To analyze the data, GraphPad Prism software v8.0 was utilized. Data were expressed as mean ± Standard Deviation (SD) and collected from each experiment in replicates. Student's *t*-test compared the difference between the two groups, and one-way ANOVA analyzed that among multiple groups; p < 0.05 was considered statistically significant.

## Results

### XBJ improves ALI

**Fig. 1:**
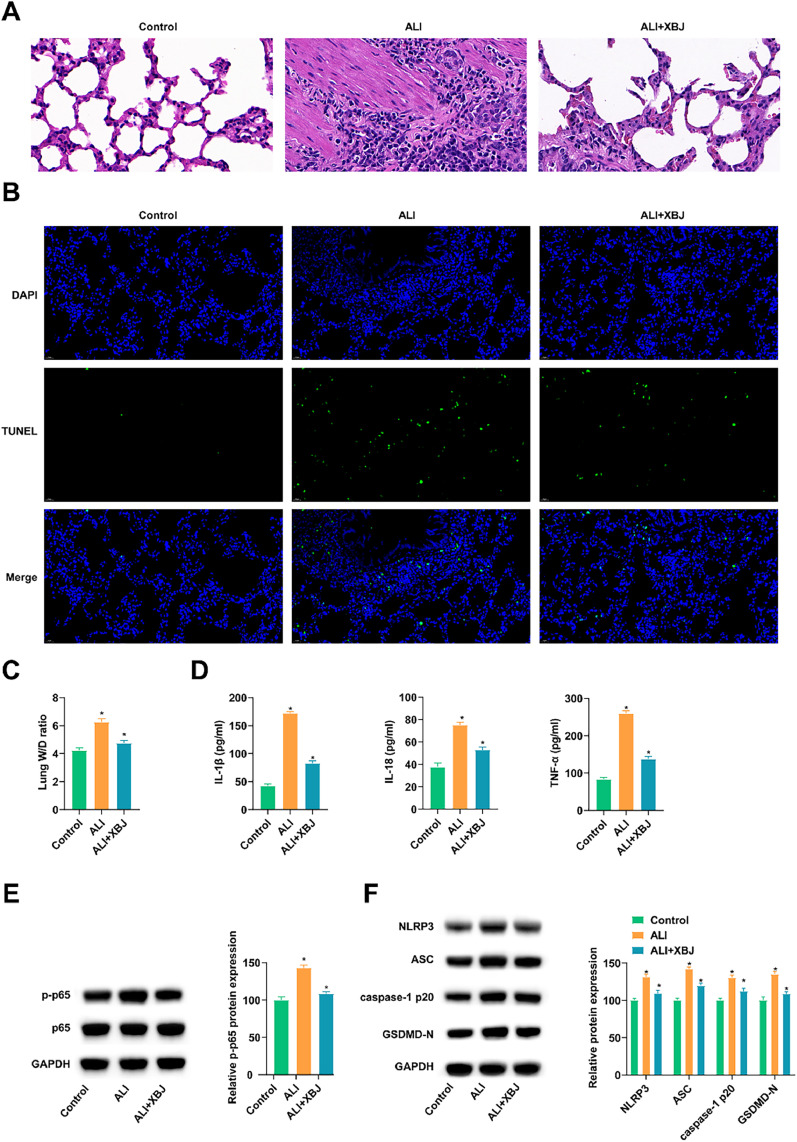
XBJ improves ALI. (A) HE-staining evaluated the morphological changes of lung tissue. (B) TUNEL staining observed the apoptotic cells in lung tissue. (C) Lung W/D ratio. (D) ELISA measured inflammatory cytokines in lung tissue. (E‒F) Immunoblotting tested p-p65, NLRP3, ASC, caspase-1 p20, and GSDMD-N. Data expressed as mean ± SD (n = 3). * p < 0.05.

LPS inducer was used to establish ALI rat models. Subsequently, HE-staining evaluated the morphological changes in the lung tissue. ALI rats showed obvious pathological injury, thickened alveolar wall diaphragm, shrunk alveolar cavity and inflammatory cells infiltrated in the lung tissue. However, the lung tissue structure of ALI rats pretreated with XBJ was intact, and the degree of inflammatory cell infiltration was low (Fig. 1A). Meanwhile, TUNEL staining showed that XBJ reduced the number of lung tissue apoptosis induced by LPS (Fig. 1B). The lung W/D ratio was increased in ALI rats, and XBJ improved LPS-induced lung injury (Fig. 1C). ELISA results demonstrated that XBJ preconditioning inhibited LPS promotion of inflammatory cytokines IL-1β, IL-18 and TNF-α in rat lung tissue (Fig. 1D). Meanwhile, immunoblotting assayed that cellular inflammation (p-p65) and pyroptosis-related proteins (NLRP3, ASC, caspase-1 p20, and GSDMD-N) were elevated in the lung tissues of ALI rats, while XBJ pretreatment could inhibit these proteins (Fig. 1 E, F).

### XBJ improves LPS-induced lung cell inflammation and pyroptosis

**Fig. 2:**
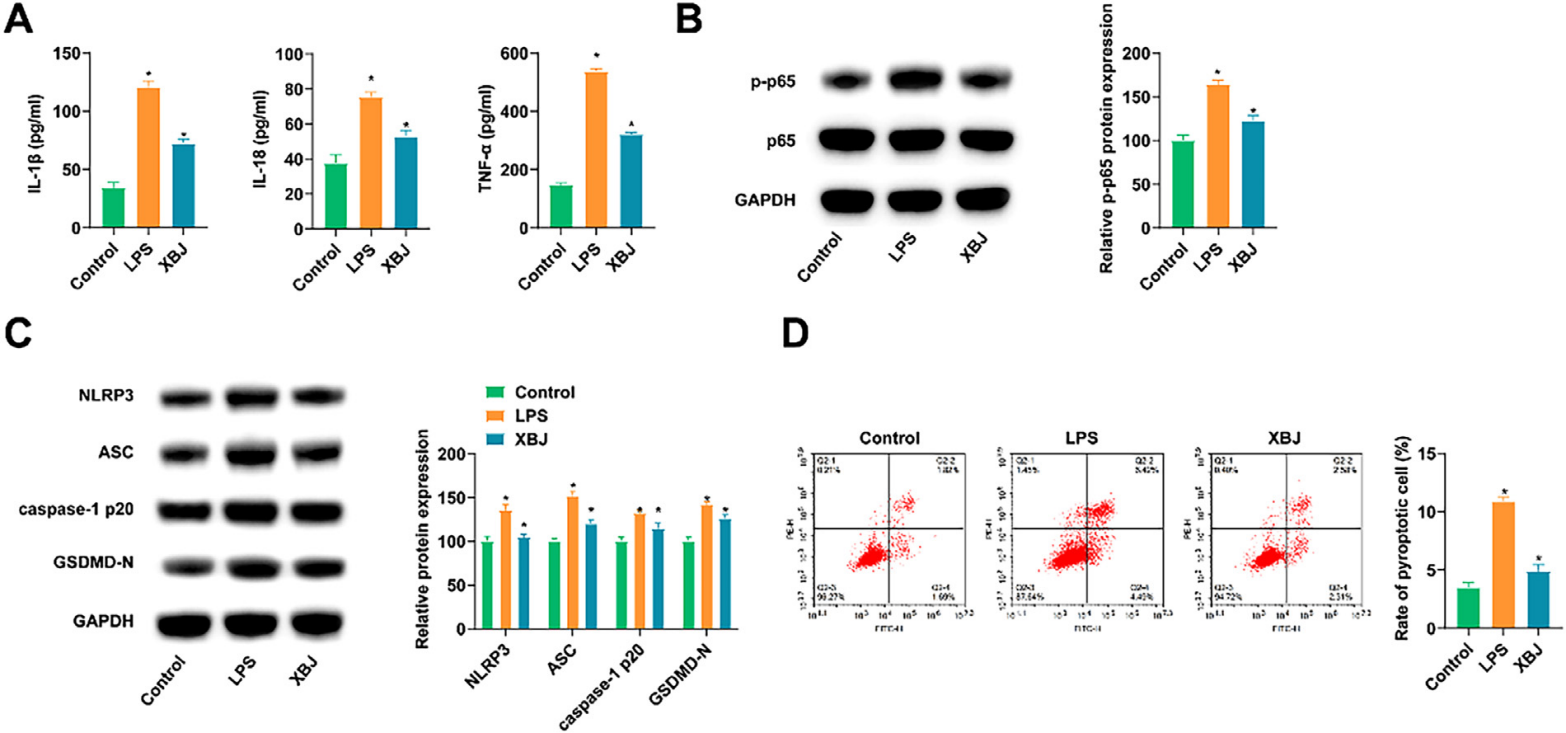
XBJ improves LPS-induced lung cell inflammation and pyroptosis. (A) ELISA measured IL-1β, IL-18 and TNF-α in cell supernatant. (B‒C) Immunoblotting tested proteins related to inflammation and pyroptosis. (D) Flow cytometry determined pyroptosis. Data expressed as mean ± SD (n = 3). * p < 0.05.

LPS-induced lung epithelial BEAS-2B cells were also used to simulate ALI. ELISA results revealed that LPS stimulated the release of inflammatory cytokines, while XBJ treatment effectively reduced inflammation in LPS-induced BEAS-2B cells (Fig. 2A). Meanwhile, immunoblotting determined that XBJ could impair LPS' promoting effect on inflammatory and pyroptosis-associated proteins in BEAS-2B cells (Fig. 2 B, C). Flow cytometry elucidated that the proportion of Caspase-1 and PI-positive cells in LPS-treated BEAS-2B cells was promoted, while XBJ treatment inhibited apoptosis (Fig. 2D).

### miR-181d-5p inhibits LPS-induced inflammatory response and pyroptosis of lung epithelial cells

**Fig. 3:**
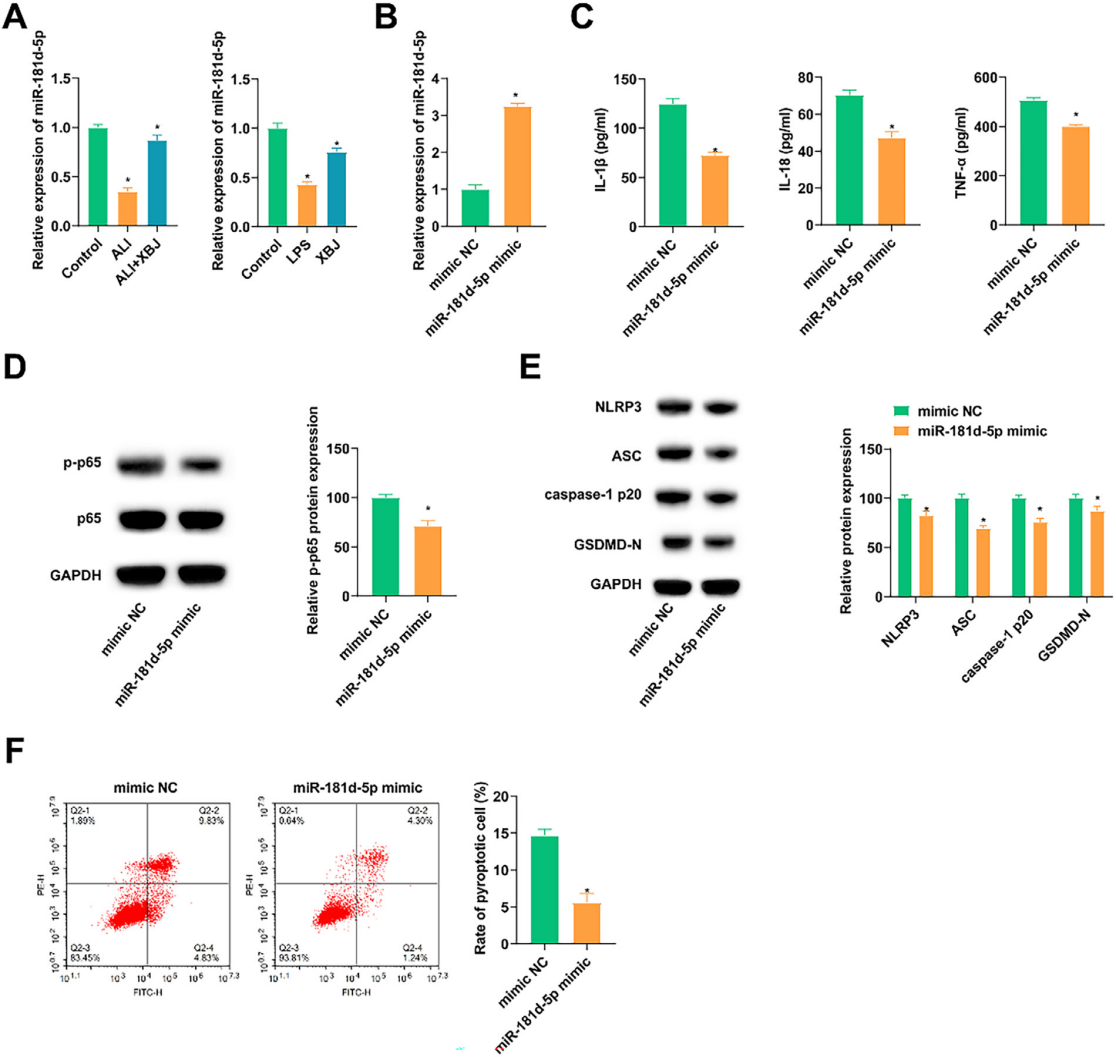
XBJ improves LPS-induced lung cell inflammation and pyroptosis. (A) RT-qPCR analyzed miR-181d-5p in LPS-treated rats and BEAS-2B cells. (B) RT-qPCR detected miR-181d-5p after transfecting miR-181d-5P mimic into LPS-induced BEAS-2B cells. (C) ELISA measured IL-1β, IL-18 and TNF-α in cell supernatant. (D‒E) Immunoblotting tested proteins related to inflammation and pyroptosis. (F) Flow cytometry determined pyroptosis. Data expressed as mean ± SD (n = 3). * p < 0.05.

miR-181d-5p was abnormally downregulated in ALI rats and BEAS-2B cells, while XBJ restored its expression pattern (Fig. 3A). Subsequently, miR-181d-5p expression was artificially modified in LPS-induced BEAS-2B cells. In detail, miR-181d-5p mimic was transfected into LPS-induced BEAS-2B cells, leading to the upregulation of miR-181d-5p (Fig. 3B). After elevating miR-181d-5p, it was measured that contents of inflammatory cytokines in the supernatant of cells were inhibited (Fig. 3C), as well as protein expression of inflammatory and pyroptosis-related proteins (Fig. 3D, E) and apoptosis rate (Fig. 3F).

### XBJ elevates miR-181d-5p and improves LPS-induced pyroptosis of lung epithelial cells

**Fig. 4:**
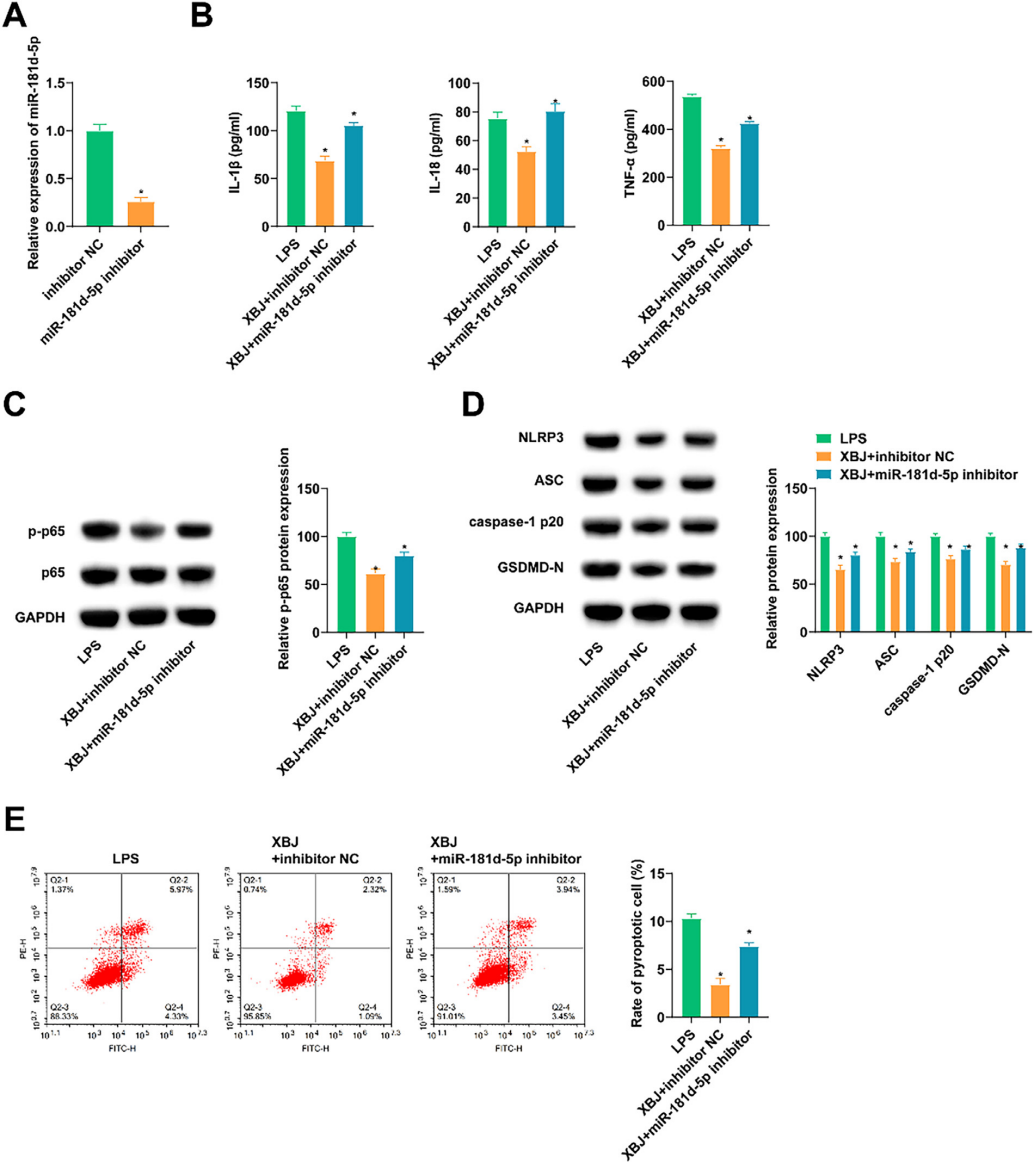
XBJ upregulates miR-181d-5p and improves LPS-induced pyroptosis of lung epithelial cells. (A) RT-qPCR detected miR-181d-5p in LPS-induced BEAS-2B cells. (B) ELISA measured IL-1β, IL-18 and TNF-α in cell supernatant. (C‒D) Immunoblotting tested proteins related to inflammation and pyroptosis. (E) Flow cytometry determined pyroptosis. Data expressed as mean ± SD (n = 3). * p < 0.05.

miR-181d-5p was silenced in BEAS-2B cells. RT-qPCR found a decrease in miR-181d-5p expression after transfection with miR-181d-5p inhibitor (Fig. 4A). The transfected cells were then treated with XBJ and LPS. Experimental data presented that XBJ inhibited inflammation and pyroptosis in LPS-induced BEAS-2B cells, but this phenomenon was counteracted after inhibiting miR-181d-5p (Fig. 4B, E).

### miR-181d-5p targets SPP1

**Fig. 5:**
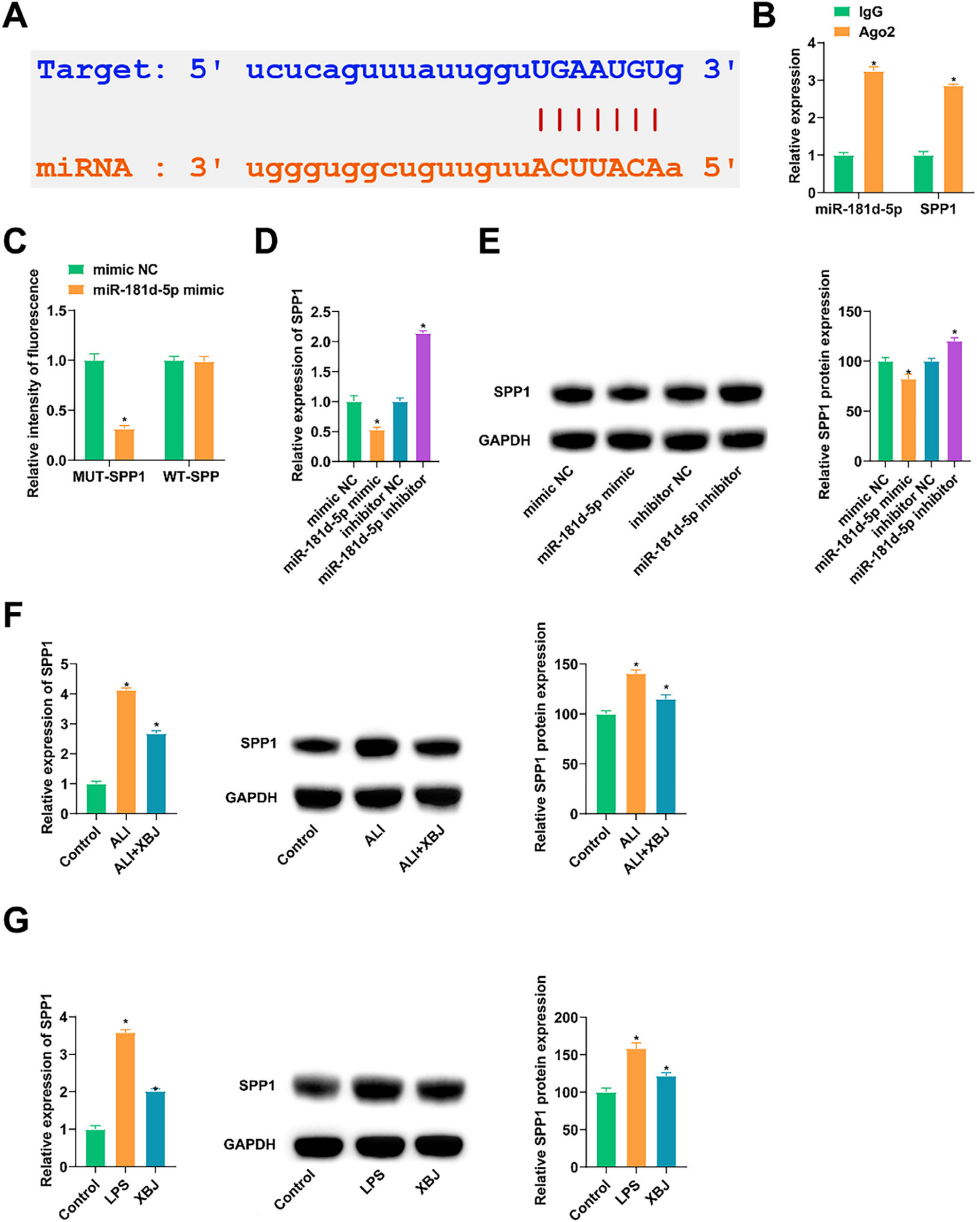
SPP1 is mediated by miR-181d-5p. (A) Potential binding sites of miR-181d-5p and SPP1. (B) RIP detected the enrichment of miR-181d-5p and SPP1 in Ago2. (C) Dual luciferase reporting experiment verified the relationship between miR-181d-5p and SPP1. (D‒E) After transfecting miR-181d-5p mimic or miR-181d-5p inhibitor into BEAS-2B cells, RT-qPCR and immunoblotting measured SPP1 expression levels. (F‒G) RT-qPCR and immunoblotting measured SPP1 in ALI rat lung tissues and BEAS-2B cells. Data expressed as mean ± SD (n = 3). * p < 0.05.

starbase3.0 (https://starbase.sysu.edu.cn/) predicted miR-181d-5p and SPP1 potential binding sites (Fig. 5A). RIP results showed that miR-181d-5p and SPP1 proteins were enriched in Ago2 (Fig. 5B). In dual luciferase reporter experiments, when co-transfected miR-181d-5p mimic and WT-SPP1, the luciferase activity decreased significantly (Fig. 5C). After transfecting miR-181d-5p mimic or miR-181d-5p inhibitor into BEAS-2B cells, SPP1 expression was suppressed or promoted (Fig. 5 D, E). Meanwhile, SPP1 was examined to be abnormally increased in ALI rats and lung epithelial cells, and XBJ inhibited its expression (Fig. 5F, G).

### XBJ upregulates miR-181d-5p and inhibits SPP1 to protect lung epithelial cells from LPS-induced injuries

**Fig. 6:**
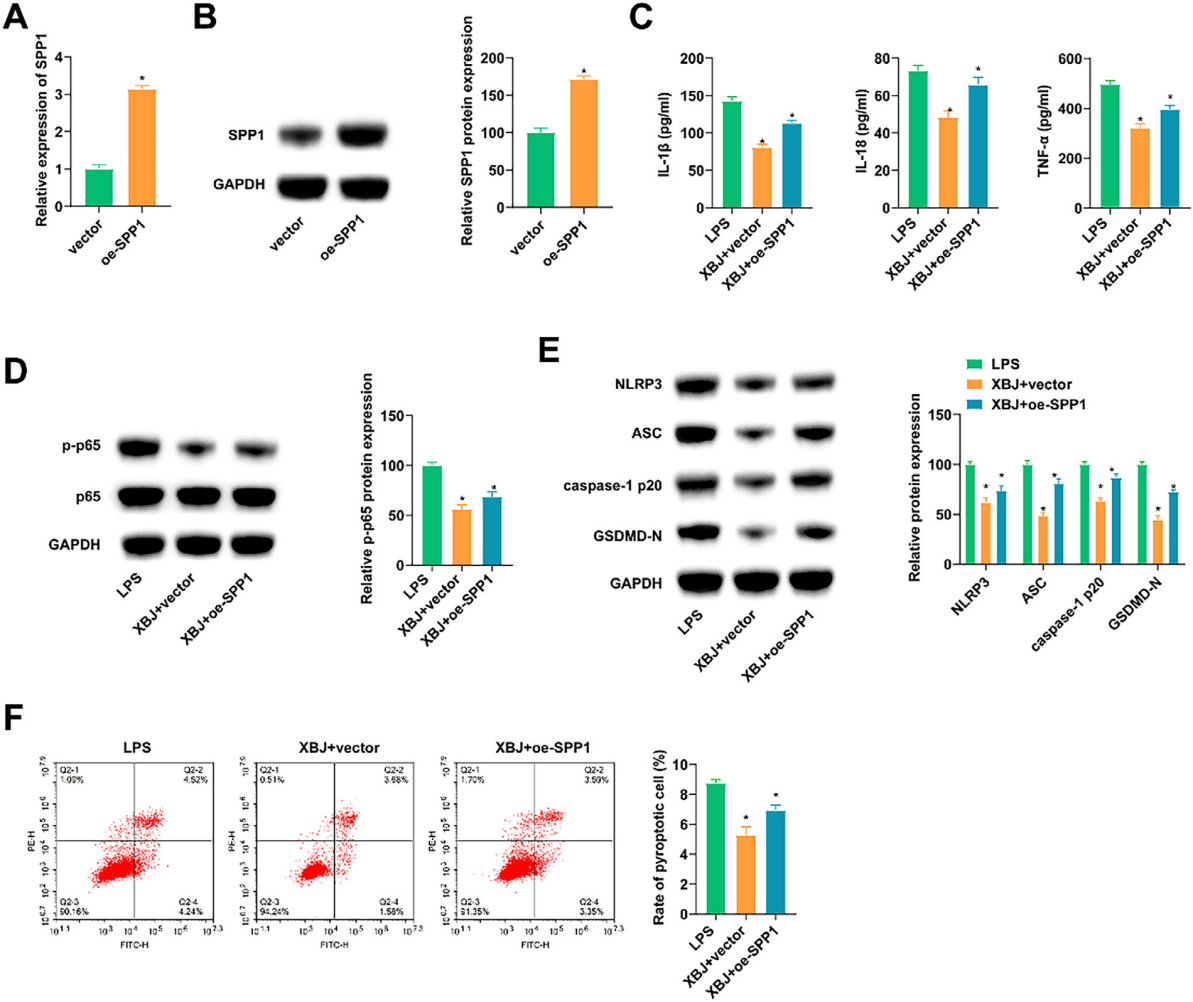
XBJ upregulates miR-181d-5p and inhibits SPP1 to protect lung epithelial cells from LPS-induced injuries. oe-SPP1 was transfected into BEAS-2B cells. (A‒B) RT-qPCR and immunoblotting measured SPP1. (C) ELISA measured IL-1β, IL-18 and TNF-α in cell supernatant. (D‒E) Immunoblotting tested proteins related to inflammation and pyroptosis. (F) Flow cytometry determined pyroptosis. Data expressed as mean ± SD (n = 3). * p < 0.05.

The oe-SPP1 was transfected into BEAS-2B cells, which successfully overexpressed SPP1 in cells (Fig. 6 A, B). Cells transfected with oe-SPP1 were treated with XBJ and LPS. Functional experiments noted that overexpressing SPP1 weakened the inhibition of XBJ on LPS-induced inflammation (Fig. 6 C, D). Meanwhile, increased expression of SPP1 antagonized the inhibition of XBJ on LPS- and ATP-induced pyroptosis (Fig. 6 E, F).

## Discussion

ALI is a respiratory disease caused by multiple factors, which may develop into acute respiratory distress syndrome [28]. A previous double-blind randomized trial demonstrated that XBJ protects lung injury by reducing neutrophil infiltration by downregulating inflammatory mediators [29]. However, the protective effect of XBJ on lung function impairment caused by ALI remains largely unknown. This study sought to explore the mechanism of XBJ in ALI and eventually confirmed that XBJ could improve ALI by mediating the miR-181d-5p/SPP1 axis to inhibit ALI inflammation and pyroptosis.

XBJ can ameliorate increased lung permeability and inflammatory response caused by sepsis [30,31]. This study constructed rat and cellular ALI models using LPS to investigate XBJ function. XBJ has been reported to protect septic ALI by inhibiting inflammation and apoptosis [32]. This is consistent with the present findings that XBJ can inhibit apoptosis and inflammation induced by ALI. But more importantly, the study also found that XBJ inhibited NLRP3, ASC, caspase-1, p20, and GSDMD-N levels, and could reverse LPS promotion of the proportion of Caspase-1 and PI-positive cells in BEAS-2B cells. Further experiments confirmed that XBJ could improve ALI by mediating the miR-181d-5p/SPP1 axis to inhibit ALI inflammatory response and pyroptosis.

In addition, since the discovery of miRNA in 1993, changes in miRNA expression have been associated with the pathogenesis of inflammatory lung diseases, making them biomarkers for novel diagnosis and treatment [33,34]. miR-181d family suggests importance in inflammation and cell growth [35]. miR-181d can promote TNF-α expression [36]. This work explored the role of miR-181d-5p in ALI inflammation and found that miR-181d-5p was abnormally downregulated in ALI, and restoring miR-181d-5p could inhibit inflammation caused by ALI. In addition, Liu's study confirmed that miR-223 specifically targets NLRP3, thereby inhibiting NLRP3 translation expression and affecting pyroptosis [37]. Notably, our study also confirmed that miR-181d-5p inhibited LPS-induced pyroptosis. Subsequently, the study explored and revealed that silencing miR-181d-5p could counteract the improvement effect of XBJ on inflammation and pyroptosis of ALI lung epithelial cells.

MiRNAs negatively regulate gene expression by binding to the 3′UTR of target genes [38]. Therefore, the study confirmed that miR-181d-5p targeted SPP1. SPP1 is a multifunctional protein expressed at the site of inflammation. For example, SPP1 is involved in acute and chronic neuritis[39]. and can mediate transfusion-related ALI by stimulating pulmonary neutrophilic accumulation [40]. This study also confirmed that SPP1 expression was elevated in ALI, and promoting SPP1 could block the protective effect of XBJ on ALI. That is, SPP1 overexpression enhanced inflammation and pyroptosis.

Some limitations exist in this study. For example, whether XBJ mediates miR-181d-5p/SPP1 axis to improve ALI was not explored in animal models of ALI, and the molecular mechanism of SPP1 regulating inflammatory response and pyroptosis has not yet been studied.

## Conclusion

XBJ improves LPS-induced ALI and inhibits LPS-induced inflammation and pyroptosis. XBJ is protective for lung cells by upregulating miR-181d-5p, thereby inhibiting SPP1. Generally speaking, XBJ provides a new therapeutic target and strategy for the clinical treatment of ALI.

## Availability of data and materials

The datasets used and/or analyzed during the present study are available from the corresponding author upon reasonable request.

## Ethical statement

All animal experiments complied with the ARRIVE guidelines and performed in accordance with the National Institutes of Health Guide for the Care and Use of Laboratory Animals. The experiments were approved by the Institutional Animal Care and Use Committee of the Affiliated Danzhou People's Hospital of Hainan Medical University.

## Funding

1. Health Industry Research Project of Hainan Province, title: Study on the protective mechanism of Xuebijing on acute lung injury induced by Endotoxin in rats based on inflammatory response and cell pyrodeath (21A200356).
2. Hainan Provincial Science and Technology Department Natural Science Foundation (High-level Talent Project) project, title: Screening intestinal flora of high-risk population of colorectal cancer in Hainan and studying its tumor-causing mechanism (822RC875).

## Declaration of competing interest

The authors declare no conflicts of interest.
